# First Permanent Molar Caries and Oral Health Practices in Saudi Male Teenagers: Inequalities by Socioeconomic Position

**DOI:** 10.1155/2020/2640949

**Published:** 2020-08-15

**Authors:** Eman Bakhurji, Balgis Gaffar, Muhammad Nazir, Khalifa Al-Khalifa, Asim Al-Ansari

**Affiliations:** Department of Preventive Dental Sciences, College of Dentistry Imam Abdulrahman Bin Faisal University, Dammam, Saudi Arabia

## Abstract

**Objectives:**

Caries remains a problem in high-risk groups aggravated by socioeconomic inequalities. The study aimed to investigate (1) oral health practices associated with caries in the 1^st^ permanent molars in Saudi male teenagers and (2) the impact of socioeconomic position (SEP) on caries control using these practices.

**Methods:**

A cross-sectional study targeted 1137 male teenagers in intermediate schools in Khobar and Dammam, Saudi Arabia, in 2016. Caries was examined using the World Health Organization criteria and plaque was assessed using the plaque index of Loe and Silness. A questionnaire assessed SEP (parental education, employment, and home ownership) and oral health practices (using fluoridated toothpaste twice daily, regular dental visits for check-ups, and avoiding daily sugary snacks). Regression models analysed the association of these factors with caries presence and the mean number of decayed 1^st^ permanent molars. Stratification was used to assess differences between SEP levels.

**Results:**

The response rate was 81.7%. Caries prevalence and mean (SD) number of decayed 1^st^ permanent molars were 50.4% and 1.08 (1.31). The use of fluoride toothpaste was associated with lower odds of caries and fewer decayed molars (OR = 0.50 and regression coefficient = −0.35). Differences in the relationship between caries and toothpaste were observed by SEP levels with stronger associations in less advantaged groups.

**Conclusions:**

Brushing twice daily using fluoride toothpaste was associated with less caries in Saudi male teenagers with stronger association observed in groups with lower SEP. The use of fluoride toothpaste helps in reducing health inequalities associated with SEP.

## 1. Introduction

The early eruption and anatomic features of the 1^st^ permanent molar predispose it to dental caries [1]. Because of its importance for normal occlusion and mastication, caries in this molar accentuates subsequent problems of dental pain, phobia, financial burden, and reduced quality of life [2–4]. There is a variation in the estimates of caries prevalence and severity in this molar among children. Tagoo et al. found that 66.4% of 1^st^ permanent molars had caries in 7–10 years school boys in Abha, Saudi Arabia [5], whereas 30.6% of 8–12-year-old Pakistani children [6] and 13.7% of 12-year-old Indian children [7] had caries in their 1^st^ permanent molars. On the other hand, Al-Samadani and Ahmad recorded caries in all four 1^st^ permanent molars in 6% of 9–12-year-old children in Jeddah, Saudi Arabia [1].

Oral health practices such as using fluoridated toothpaste, consuming sugary snacks, and visiting dentists for routine dental check-ups were reported to influence caries prevalence in children [8, 9]. Prevalence and severity of caries were also affected by socioeconomic position (SEP) [10]. Differences between social classes lead to health inequalities with greater amount of disease and more severe forms of it occurring in those from low SEP [11, 12]. Several SEP measures were used in studies investigating its impact on oral health showing that SEP is a complex construct that acts in different ways and has various health effects. Parents' education, especially that of the mother, employment, and home ownership were strong indicators of SEP and have been reported to affect health, in particular oral health [13–15].

Saudi Arabia provides a setting to study SEP inequalities in health and the adoption of oral health practices. The country's economy and literacy rate contrast with the presence of employability and housing problems [16]. Caries prevalence is consistently reported to be high in Saudi Arabia [17–19] and oral health practices to be suboptimal [20, 21]. It would be useful to investigate how these different SEP influences might impact inequalities in caries and in the adoption and effectiveness of dental preventive practices. This helps in shaping health policy and promoting self-care in Saudi Arabia and similar countries where the implementation of community-wide strategies like water fluoridation might not be feasible [22, 23]. In this case, individual oral health practices might be the main tool to control caries. The aims of the present study were (1) to assess oral health practices associated with caries in the 1^st^ permanent molars in male teenagers in Saudi intermediate schools and (2) to assess the impact of SEP inequalities on caries control using oral health practices adopted by the individual.

## 2. Materials and Methods

The present cross-sectional study was conducted in intermediate schools in Dammam and Khobar, Eastern Province, Saudi Arabia. The current report is part of a larger study that aimed to describe the oral health status of intermediate school students. The target group was male teenagers between 12 and 15 years of age. Subjects were included if (1) they attended public intermediate schools in Dammam and Khobar, (2) their parents provided written consent for their children's participation and the student assented to be clinically examined, and (3) they had no known history of systemic diseases or recent (within the last 6 months) medication use based on information provided by parents.

The sample size of the original study was estimated using the following assumptions: alpha error = 5%, study power = 90%, estimated caries prevalence = 70%, and null percent = 65%. The minimum required sample size was calculated to be 927 (https://www.stat.ubc.ca/∼rollin/stats/ssize/b1.html). The sample size was increased to 1,020 to make up for an estimated nonresponse rate = 10%. A formal request was made to the Directorate of Education in the Province to visit intermediate schools to recruit the required number of schools. The Directorate randomly assigned three schools for the study. All teenagers in the three schools were invited to join the study if they fulfilled the inclusion criteria (*n* = 1392). Ethical approval was obtained from the Institutional Review Board at the University of Dammam (IRB-2015-02-187). The study was conducted in accordance with the Helsinki declaration.

Data collection included a clinical examination to measure caries and plaque accumulation. Three examiners were calibrated to a gold standard examiner with acceptable interexaminer reliability (Kappa ≥0.6). They performed the clinical examination in the schools, using disposable mirrors in classroom daylight with the student seated on a chair. Caries was diagnosed at the cavitation level and recorded using the World Health Organization criteria with visual examination where the probe was used mainly to remove food remains and to ascertain softness of the floor when needed [24]. The plaque was measured using the Plaque Index of Loe and Silness by passing a blunt probe on the surfaces of 6 index teeth (upper right first molar, upper right lateral incisor, upper left first premolar, lower right first premolar, lower left lateral incisor, and lower left first molar). Plaque accumulation was recorded at two points on each of the buccal and lingual surfaces and the four readings averaged for each tooth followed by averaging the readings of the six teeth to obtain the plaque index of the student [25].

A questionnaire was designed based on previous studies and modified to fit the study objectives [10, 13, 14, 26]. It was assessed for face and content validity by two independent reviewers and pilot-tested for clarity among 15 teenagers before the study. The questionnaire consisted of two sections. The first section collected information about the socioeconomic background including parental education (university educated or not) and employment status (fathers; retired/not employed, employed in the public or private sector, mothers; employed or housewife) and home ownership (rented or owned house). Parents' employment was later categorized into both parents employed versus not. The second section included questions assessing oral health practices (whether the respondent used fluoridated toothpaste twice daily, had regular visits to the dentists for check-ups, and avoided daily sugary snacks). The questionnaire was sent home with the child to be filled by the parents. It was collected by the teacher and handed to the study team before the examination visit.

The analysis was performed using SPSS version 20.0 at 5% significance level. Two main outcomes were measured in this study for the 1^st^ permanent molar: caries prevalence (present/absence) and caries severity (number of carious molars). The following regression models were developed:

1. First set: The outcome was caries in the 1^st^ permanent molars (including prevalence and severity) and the independent variables included oral health practices and socioeconomic background. The association was assessed in univariate regression models (logistic for presence of caries and linear for the number of carious 1^st^ molars). Variables with a significant association with the outcomes were entered into multivariable regression models.
2. Second set: The outcome was caries in the 1^st^ permanent molar (prevalence and severity) and the independent variables were factors with a significant association in the first set stratified by SES levels to assess caries inequalities.

## 3. Results

Out of all students invited to participate in the study, the parents of 1137 responded to the questionnaire and the students were clinically examined (response rate = 81.7%). Half of the fathers (*n* = 561) and 42.5% (*n* = 469) of the mothers were university educated. Half of the fathers (*n* = 516) were employed in the public sector and 73.3% (*n* = 789) of the mothers were housewives. About one-quarter (*n* = 272) of teenagers had both parents employed and 53.9% (*n* = 589) lived in owned houses. Most teenagers were reported to use fluoridated toothpaste (*n* = 224, 60.4%), not have regular visits to the dentist (*n* = 279, 74.8%), and consume sugary snacks daily (*n* = 777, 75.9%). The mean (SD) plaque index was 1.4 (0.9) (Table 1).

The overall prevalence of tooth decay was 57.2% (*n* = 650) and the mean (SD) number of decayed teeth was 1.63 (1.99). Caries prevalence in the 1^st^ molar was 50.4% (*n* = 537) and the mean (SD) number of carious 1^st^ molars was 1.08 (1.31). Participants of university educated parents most commonly used fluoridated toothpaste (70.1%), had the lowest prevalence of caries (44.6% for father, 44.7% for mother), and had the lowest mean carious first molars (Table 2).

The univariate regression in Table 3 shows that plaque accumulation was associated with significantly higher odds of caries presence in the 1^st^ permanent molars (OR = 1.39), whereas higher level of father's and mother's education, using fluoridated toothpaste, and having regular dental visits were associated with significantly lower odds (OR = 0.65, 0.77, 0.49, and 0.74, respectively). Greater number of decayed 1^st^ permanent molars was associated with nonemployed fathers and plaque index (regression coefficients = 0.30 and 0.27, respectively), whereas a lower number of decayed molars were associated with having university educated fathers, using fluoridated toothpaste and regular visits to the dentist (regression coefficient = −0.30, −0.43 and −0.23, respectively). In multivariate regression, only using fluoridated toothpaste was associated with lower odds of caries presence (OR = 0.50) and fewer decayed 1^st^ molars (regression coefficient = −0.35).

Table 4 shows the association between caries prevalence and severity and using fluoridated toothpaste stratified by SEP levels. Caries odds when using fluoridated toothpaste were similar whether fathers were university educated or not (0.52 and 0.48, respectively). Greater differences were observed in the odds ratios in children of mothers who were university educated or not (OR = 0.97 and 0.36, respectively), when both parents were employed or not (OR = 0.59 and 0.46, respectively) and if houses were owned or rented (OR = 0.67 and 0.35, respectively). The same pattern was observed in the regression coefficients of the number of carious 1^st^ molars associated with using fluoridated toothpaste that were lower in lower socioeconomic position. The regression coefficient of non-university educated fathers was lower than that of university educated fathers (coefficients = −0.50 and −0.32), and so was the coefficient for non-university educated and university educated mothers (−0.51 and −0.09), not both parents employed and both parents employed (−0.51 and −0.18), and living in rented and owned houses (−0.63 and −0.21).

## 4. Discussion

Our study showed that brushing with fluoridated toothpaste was significantly associated with lower odds of caries in the 1^st^ permanent molars of Saudi male teenagers after adjusting for the effect of SEP, other oral health practices, and plaque. Although SEP factors were not significantly associated in multivariate regression with caries, they affected the magnitude of the association between using fluoridated toothpaste and caries with stronger association existing among those from lower SEP.

In the present study, 50% of male teenagers had caries in the 1^st^ permanent molars. This prevalence was lower than that reported in Abha, KSA (66.4%) [5], and higher than in Jeddah, KSA (6%) [1]. The mean number of decayed 1^st^ permanent molars in the present study was 1.08, which is higher than that observed by Tagoo et al. in Abha (2.74) [5]. Our study found that 60.4% of participants brushed their teeth twice daily using a fluoridated toothpaste similar to that reported by Al-Subait et al. (66.5%) among 10–18 years old in Riyadh [27]. The level of tooth brushing reported in our study, however, was much higher than that reported among 12–14-year-old children in Qatar (3.7%) [28]. In our study, 25% of teenagers reported regular check-ups. Our results agree with other studies among Saudi children reporting dental visits mostly on pain including 75% of 10–18-year-old children attending an event in Riyadh visited the dentist during the last six months with 50% doing so because of pain [27] and 46% of 9–12-year-old children from Madinah who visited the dentists only when necessary) [29]. Our study reported that 24.1% of participants did not consume sugary snacks daily which generally agrees with a level of 35.8% and 31.6% of intermediate school children in Riyadh reported to rarely consume sugary snacks and drinks, respectively [30].

Our study used twice daily fluoridated toothpaste in the analysis because this frequency of tooth brushing is effective against caries [31]. The findings of our study indicate that there is a stronger relationship between fluoridated toothpaste and caries prevention among the less advantaged than those from higher social classes. These results differ from those of the study by Ellwood et al. in England [32]. They found no reduction in caries inequalities associated with deprivation after using fluoridated toothpaste and the absolute difference was 0.5 DMFT in the most and least deprived quartiles. In contrast, Sabbah et al. reported that caries incidence rate ratio (IRR) in Finnish adults of basic and secondary education over 4 years was 2.23 and 1.01 compared to 1 in adults with higher education [33]. Among those who reported using fluoridated toothpaste in that study, a reduction of 24% in IRR occurred in the basic education group compared to 5% increase in the secondary education group. The authors concluded that fluoridated toothpaste can reduce health inequalities among Finnish adults. Caution is warranted when comparing our results to theirs due to the different age groups in both studies and the egalitarian nature of the Finnish healthcare system which might further reduce inequalities in access to care for low SEP groups.

In the present study, there was a greater selective preventive benefit of fluoridated toothpaste to lower social levels and this was observed when the prevalence of its use in low SEP groups was similar to that in high SEP groups. Health interventions were reported to reduce rather than increase inequalities depending on their rate of diffusion and adoption [34]. If being of higher SEP makes no difference in the use of these interventions, then the effect of social status becomes less important and inequalities are reduced [35]. In a clinic-based study including Saudi adults in Dammam, Saudi Arabia, Al-Ansari reported that using preventive methods in general (including tooth brushing with fluoridated toothpaste) was not significantly associated in multivariate regression with the subjects' educational level or occupation [36]. If the use of fluoridated toothpaste was minimally associated with SEP and it was equally available to less privileged groups, this would help reduce inequalities and might explain our results. Developing pricing policies that ensure the availability of preventive products to different SEP levels might help in further reduction of caries inequalities [36]. Another factor that might affect the impact of toothpastes in reducing health inequalities is awareness [37, 38]. In our study, parental education was the SEP factor where inequalities were mainly observed. This agrees with the findings of a study of adult Saudis visiting a university clinic where awareness significantly affected the use of preventive methods [35]. Education is free for Saudi citizens at all levels with 94.68% literacy rate and 30.2% higher education enrolment ratio. Participants' responses about parental education appear close to what exists in Saudi Arabia and reported in a previous study from Dammam [39]. Increasing the awareness of teenagers and the community, in general, especially those with low SEP, would promote the use of fluoridated toothpaste and might reduce caries inequalities. The SEP differences observed in our study might be partly due to other caries preventive factors that the more advantaged groups could have been using which would reduce the significance of fluoridated toothpaste on its own. These may include the use of professional fluoride applications or fluoride home care products so that the effect of toothpaste was less critical than it would be in the low social classes.

Saudi Arabia is a high-income country with a gross domestic product per capita of $22,865 in 2019. Top international brands of toothpastes are freely available in Saudi Arabia and the price of these brands of toothpaste (100 mg) ranges from 6 Saudi riyals (SAR) to 25 SAR (1 SAR equivalent to 0.27 USD). Most people can afford easily available toothpaste in the country. Therefore, the availability and price of toothpaste might not influence study results.

A previous study of Danish adolescents evaluated associations between socioeconomic position and caries experience using covariates such as type of household, number of family members, immigration status, and country of origin [40]. Similarly, ethnic background and socioeconomic position measured by family, school, and residential deprivation of children were used in another study to assess their influence on decayed teeth [41]. On the other hand, the present study used parental education, employment status, and home ownership to measure SEP. Although Saudi Arabia has considerable population of immigrants, however, their children mostly study in private or embassy schools of respective countries. Public schools in Saudi Arabia consist of Saudi children. Since the present study was conducted in public schools, therefore, information on immigration and country of origin was not used in the study. The influence of the number of children and family members on caries experience should be considered in future studies.

The limitations of our study are related to the inclusion of only male teenagers. The sample was not statistically selected and was restricted only to public schools assigned by the Directorate of Education in the Province. Because of this, there is a possibility of selection bias; hence, generalization on a wider scale should be undertaken with caution. Researchers used different categories of SEP (parental education (3 categories), income (4 categories), and occupational social class (4 categories) [40]. However, fewer categories were used in the present study which may lead to biases in the results. In addition, the oral health practices in our study were self-reported with potential social desirability bias where favourable practice such as brushing may be overreported and negative practices such as consuming sugary snacks may be underestimated [42]. DMFT index is generally used to evaluate caries experience by including decayed (D), missing (M), and filled (F) teeth; however, the present study recorded caries as decay because DMFT index does not directly indicate the intensity of the carious attack. In addition, DMF data are of little value for assessing caries treatment needs. Literature indicates the use of decayed teeth to measure clinical oral health outcomes and filled teeth to evaluate access to oral care and receipt of caries treatment [41]. Our study was cross-sectional which means that the associations should not be interpreted as causative except after confirmation by future longitudinal prospective studies.

## 5. Conclusions

Using fluoridated toothpaste twice daily was associated with lower odds of caries and fewer carious 1^st^ permanent molar in Saudi male teenagers. It was also associated with reducing health inequalities between different SEP levels when groups from lower and higher SEP used it at similar levels. Healthcare planners are advised to promote teenagers' use of fluoridated toothpaste to reduce caries inequalities attributed to SEP.

## Figures and Tables

**Table 1:** Distribution of socioeconomic factors and oral health practices in teenagers.

Variables		Frequency (%)
Father's education	University educated	561 (50.6)
	Not university educated	548 (49.4)
Mother's education	University educated	469 (42.5)
	Not university educated	634 (57.5)
Father's employment	Not employed/retired	112 (10.5)
	Private sector	444 (41.6%)
	Public sector	516 (48.1)
Mother's employment	Housewife	789 (73.3)
	Employed	287 (26.7)
Both parents employed	Yes	272 (23.9)
	No	865 (76.1)
Home ownership	Rented	503 (46.1)
	Owned	589 (53.9)
Using fluoridated toothpaste twice daily	Yes	224 (60.4)
	No	147 (39.6)
Regular visits to the dentist for check-ups	Yes	94 (25.2)
	No	279 (74.8)
Not consuming sugary snacks daily	Yes	247 (24.1)
	No	777 (75.9)
Plaque index	Mean (SD)	1.4 (0.9)

**Table 2:** Prevalence of caries and the use of fluoridated toothpaste among study participants.

		Percentage using fluoridated toothpaste (%)	Prevalence of caries (%)	Number of carious 1^st^ molars: mean (SD)
All		60.4	50.4	1.08 (1.31)
Father's education	University educated	70.1	44.6	0.91 (1.23)
	Not university educated	51.1	55.5	1.21 (1.35)
Mother's education	University educated	70.1	46.7	0.98 (1.26)
	Not university educated	54.7	53.2	1.13 (1.33)
Both parents employed	Yes	60.3	50.7	0.99 (1.21)
	No	60.9	50.3	1.11 (1.34)
Home ownership	Owned	60.6	48.2	1.03 (1.28)
	Rented	60.1	52.9	1.12 (1.32)

**Table 3:** Association of caries in 1^st^ molars with socioeconomic factors and preventive practices in male teenagers in Khobar and Dammam, 2016.

Variables		Presence of caries in 1^st^molars OR (95% C.I.)		Number of carious 1^st^ molars regression coefficient (95% C.I.)	
		Univariate	Multivariable	Univariate	Multivariable
University educated father	Yes (ref), no	0.65 (0.51, 0.82)^*∗*^	0.90 (0.49, 1.66)	−0.30 (−0.46, −0.15)^*∗*^	−0.22 (−0.56, 0.12)
University educated mother	Yes (ref), no	0.77 (0.61, 0.98)^*∗*^	0.78 (0.42, 1.44)	−0.15 (−0.30, 0.01)	
Father's employment	Retired/not employed (ref), public sector	1.32 (0.88, 1.97)		0.30 (0.04, 0.56)^*∗*^	0.35 (−0.27, 0.96)
	Private sector (ref), public sector	0.79 (0.61, 1.01)		−0.15 (−0.31, 0.01)	0.08 (−0.27, 0.43)
Mother's employment	Housewife (ref), employed	0.91 (0.70, 1.19)		0.04 (−0.14, 0.21)	
Home ownership	Rented (ref), owned	1.21 (0.95, 1.53)		0.09 (−0.06, 0.25)	
Plaque index		1.39 (1.17, 1.65)^*∗*^	1.35 (0.99, 1.84)	0.27 (0.16, 0.38)^*∗*^	0.17 (−0.02, 0.35)
Using fluoridated tooth paste twice daily	Yes (ref), no	0.49 (0.32, 0.74)^*∗*^	0.50 (0.28,0.88)^*∗*^	−0.43 (−0.69, −0.18)^*∗*^	−0.35 (−0.69, −0.004)^*∗*^
Regular visit to the dentist for check-ups	Yes (ref), no	0.74 (0.55, 0.98)^*∗*^	0.69 (0.37, 1.30)	−0.23 (−0.42, −0.04)^*∗*^	−0.19 (−0.58, 0.21)
Not consuming sugary snacks daily	Yes (ref), no	0.97 (0.73, 1.29)		0.02 (−0.17, 0.20)	

^*∗*^Statistically significant at *P* ≤ 0.05, OR: odds ratio, C.I.: confidence interval, and ref: reference category. Logistic regression used for caries presence and linear regression used for number of caries molars.

**Table 4:** Association between 1^st^ molar caries and using fluoridated toothpaste twice daily in different SEP levels in male teenagers in Khobar and Dammam, 2016.

		Association between caries in 1^st^ molar and toothpaste use	
		Presence of caries OR (95% CI)	Number of carious 1^st^ molars regression coefficient (95% CI)
All		0.50 (0.28, 0.88)^*∗*^	−0.35 (−0.69, −0.004)^*∗*^
Father's education	University educated	0.52 (0.28, 0.99)^*∗*^	−0.32 (−0.69, 0.06)
	Not university educated	0.48 (0.27, 0.87)^*∗*^	−0.50 (−0.86, −0.14)^*∗*^
Mother's education	University educated	0.97 (0.49, 1.94)	−0.09 (−0.47, 0.30)
	Not university educated	0.36 (0.20, 0.64)^*∗*^	−0.51 (−0.85, −0.16)^*∗*^
Both parents employed	Yes	0.59 (0.25, 1.39)	−0.18 (−0.67, 0.31)
	No	0.46 (0.28, 0.74)^*∗*^	−0.51 (−0.81, −0.21)^*∗*^
Home ownership	Owned	0.67 (0.37, 1.22)	−0.21 (−0.60, 0.18)
	Rented	0.35 (0.19, 0.65)^*∗*^	−0.63 (−0.98, −0.28)^*∗*^

The models were adjusted for other oral health practices (regular visits to the dentist for check-ups and avoiding daily sugary snacks) and plaque index. Logistic regression used for caries presence and linear regression used for number of caries molars.^*∗*^Statistically significant at *P* ≤ 0.05; OR: odds ratio and CI: confidence interval.

## Data Availability

The SPSS data file of this study is available from the corresponding author upon request.
